# Free-breathing myocardial T2* mapping using GRE-EPI and automatic non-rigid motion correction

**DOI:** 10.1186/1532-429X-17-S1-W8

**Published:** 2015-02-03

**Authors:** Ning Jin, Marie-Pierre Jolly, Subha V Raman, Orlando P Simonetti

**Affiliations:** 1 Siemens Healthcare, Columbus, OH, USA; 2 Siemens Corporate Research, Princeton, NJ, USA; 3 Division of Cardiovascular Medicine, Department of Internal Medicine, The Ohio State University Wexner Medical Center, The Ohio State University, Columbus, OH, USA; 4 Dorothy M. Davis Heart and Lung Research Institute, The Ohio State University, Columbus, OH, USA; 5 Department of Radiology, The Ohio State University Wexner Medical Center, The Ohio State University, Columbus, OH, USA

## Background

Myocardial T2* mapping is a widely used method to detect and quantify cardiac iron overload in transfusion-dependent anemia patients (1, 2). The ECG-triggered black-blood multi-echo gradient echo sequence typically used for myocardial T2* mapping acquires segmented k-space data across multiple heart beats (BH Seg-mGRE) (3). However, it is highly sensitive to respiratory motion and requires patient breath-hold. In this work, we present a new free-breathing technique to accurately quantify myocardial T2* by acquiring multiple, single heartbeat images insensitive to respiratory motion artifact, and applying automatic non-rigid motion correction (moco GRE-EPI) to register images prior to T2* map estimation.

## Methods

We developed a black-blood, multi-shot, GRE-EPI sequence with TR = 20 ms, echo-train-length = 5, GRAPPA acceleration rate = 2 and a water excitation pulse to minimize fat displacement artifacts. Eight images were acquired at echo times ranging from 1.2 ms to 15 ms; each T2*-weighted image was acquired in one heartbeat with an acquisition window of 240 ms and repeated with four measurements. The total acquisition duration was 64 heart beats. Automatic in-plane non-rigid motion correction (4, 5) was used to compensate for mis-registration due to respiratory motion. Motion correction (moco) was applied first across the four measurements at each TE prior to averaging, and then across the averaged images at different TEs prior to generating the T2* map. T2* maps were calculated by fitting a mono-exponential model. Both acquisition techniques were attempted in 72 patients referred for clinical CMR cardiomyopathy evaluation on the 1.5 T scanner (Avanto, Siemens). Both moco GRE-EPI during free-breathing and BH Seg-mGRE during breath-hold were acquired in a single mid-short axis plane. T2* was measured in the interventricular septum and liver.

## Results

**Figure 1:**
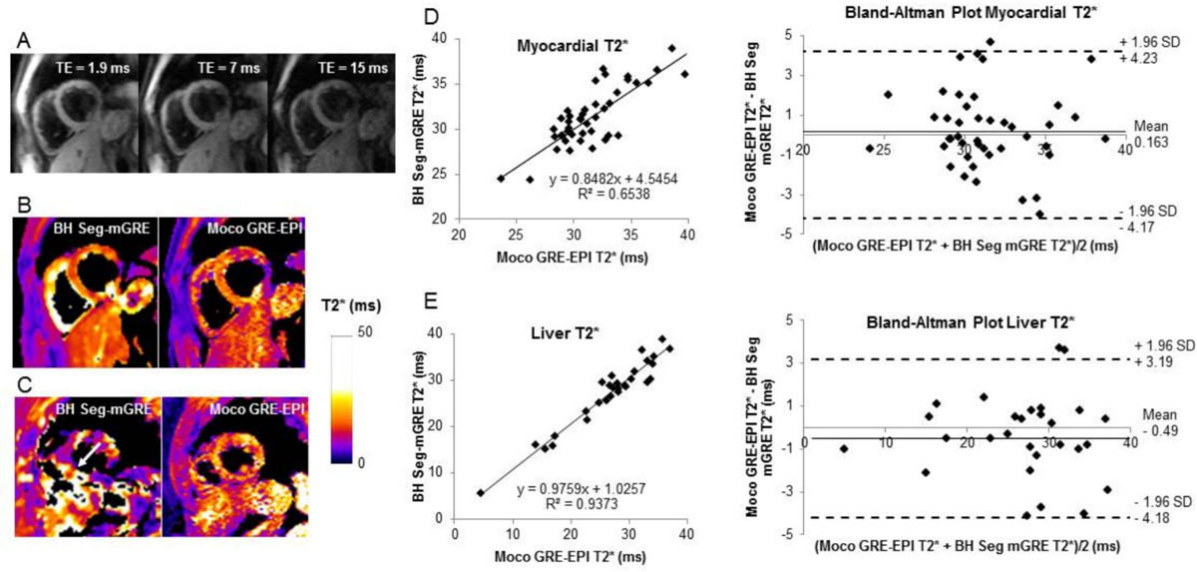
(A) motion corrected averaged T2*-weighted images at TE = 1.9, 7 and 15 ms from the moco GRE-EPI sequence. (B) One example of good T2* map quality for BH seg-mGRE and moco GRE-EPI in a patient able to breath hold. (C) In patients unable to breath hold, free-breathing moco GRE-EPI improves T2* map quality. (D, E) Comparisons between the mean T2* measured in the intraventricular septum and the liver from the two techniques demonstrated strong correlations and no significant differences.

Conventional BH Seg-GRE failed in 24 out of 72 patients due to the inability to breath-hold while the new moco GRE-EPI technique failed in only 6 patients due to the severe respiratory motion or imperfect fat suppression (Figure 1, Panels A-C). Comparisons between T2* measured in the intra-ventricular septum and the liver from the two techniques in 45 patients with adequate images using both techniques (Figure 1, panels D and E) demonstrated strong correlations (*r* = 0.77 and 0.97) and no significant differences (*p* = 0.78 and 0.24).

## Conclusions

We have described a technique for T2* mapping using moco GRE-EPI that enables accurate myocardial T2* measurements and is insensitive to respiratory motion. It could be especially beneficial for patients who are unable to breath-hold.
